# Fifteen years of epidemiology in BMC Medicine

**DOI:** 10.1186/s12916-019-1407-5

**Published:** 2019-09-23

**Authors:** Deborah A. Lawlor

**Affiliations:** MRC Integrative Epidemiology Unit at the University of Bristol, Population Health Science, Bristol Medical School and Bristol NIHR Biomedical Research Centre, Bristol, UK

**Keywords:** Epidemiology, Causality, Big data

## Abstract

*BMC Medicine* was launched in November 2003 as an open access, open peer-reviewed general medical journal that has a broad remit to publish “outstanding and influential research in all areas of clinical practice, translational medicine, medical and health advances, public health, global health, policy, and general topics of interest to the biomedical and sociomedical professional communities”. Here, I discuss the last 15 years of epidemiological research published by *BMC Medicine*, with a specific focus on how this reflects changes occurring in the field of epidemiology over this period; the impact of ‘Big Data’; the reinvigoration of debates about causality; and, as we increasingly work across and with many diverse disciplines, the use of the name ‘population health science’. Reviewing all publications from the first volume to the end of 2018, I show that most *BMC Medicine* papers are epidemiological in nature, and the majority of them are applied epidemiology, with few methodological papers. Good research must address important translational questions that should not be driven by the increasing availability of data, but should take appropriate advantage of it. Over the next 15 years it would be good to see more publications that integrate results from several different methods, each with different sources of bias, in a triangulation framework.

**Electronic supplementary material:**

The online version of this article (10.1186/s12916-019-1407-5) contains supplementary material, which is available to authorized users.

## Background

**Fig. 1:**
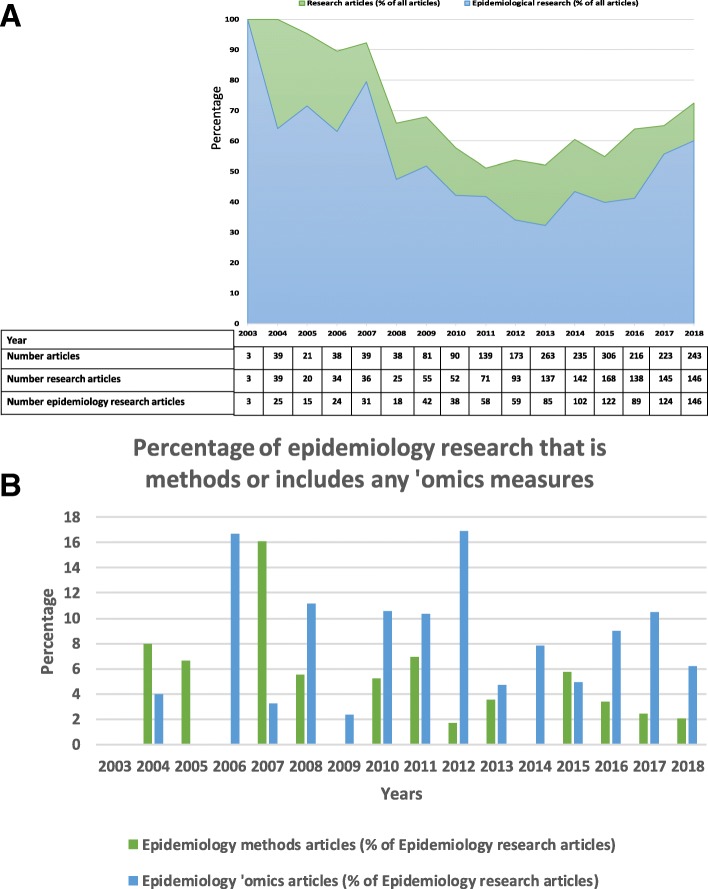
Research articles and ‘epidemiology’ research articles published in *BMC Medicine*, 2003–2018. **a** the proportion of all research articles that were epidemiology studies, by years. **b** the proportion of epidemiology study papers that were methodological or included any ‘omics measurements

In the 15 years since *BMC Medicine* was launched in November 2003, epidemiology has led the challenge of ‘Big Data’ science [1], reinvigorated debates about what can legitimately be considered causes of diseases and what methods should be used to determine causality (e.g., [2, 3]), and become increasingly known as ‘population health science’ [4]. These three changes are related to each other and to broader changes in science and society, as well as being rooted in a much longer history going back decades if not centuries. I thought it would be interesting to consider how these recent changes are reflected in the last 15 years of *BMC Medicine*. To do this, I undertook a review of the types of studies published by *BMC Medicine* in the last 15 years (see Fig. 1 and Additional file 1 for the methodology used to prepare this figure). I was pleased to see that most of the published research articles were epidemiology studies (Fig. 1a; 981/1334; 74%). Most of the epidemiology papers were applied studies (Fig. 1a; 946/981; 96%). This is a common finding in general medical journals, despite the existence of several specific epidemiology journals [5]. The few papers that I considered to be methodological (Fig. 1b; 35/981; 4%) were largely concerned with methods for developing or refining tools to measure risk factors or disease outcomes (e.g., [6, 7]), rather than research into analytical or study design methods. There was little evidence that authors were using directed acyclic graphs (DAGs) to demonstrate statistical assumptions [8].

### Big data

‘Big Data’ has no clear definition, but the term can be used to refer to datasets with many participants and/or many variables. The former category includes large-scale record linkage studies; the latter includes the integration of multiple ‘omics data with socioeconomic, environmental, lifestyle and clinical data in epidemiological studies and the collection of intense, continuously measured data, such as glucose levels collected by sensors at short, regular intervals. The current *BMC Medicine* call for papers in this area notes: “Big Data in Medicine can be used to provide health profiles and predictive models around individual patients. The use of high-throughput data to integrate genetic and clinical inter-relationships; real-world data to infer biological principles as well as associations, trajectories and stratifications of patients; data-driven approaches for patients and digital platforms are the hope for medical problems and evidence-based medicine” [9].

However, as Saracci has eloquently highlighted, excessive claims for ‘Big Data’, such as is proposed in this statement, can result in ‘bigness’ overriding the key principles of epidemiology and good science. These principles include, for example, the need for data (and software) validity, replication or validation of results in independent studies and, importantly, using data to address the most relevant questions rather than ‘blind [big] data dredging’ [1]. As with other journals, *BMC Medicine* has published a small proportion of ‘omics studies (Fig. 1b; 77/981 (8%) of the epidemiology papers included some ‘omics measurements) and most of these were small and had no independent replication or validation (e.g., [10–12]). Larger studies that did include replication (e.g., [13, 14]) have been published more recently.

### Population health science

The increasing use of the term ‘population health science’ in part reflects the potential for epidemiologists to undertake population level physiology and embed this in what was previously called ‘social medicine’. This is enabled by the integration of multiple ‘omics data with socioeconomic, lifestyle and clinical data in large cohort studies. Multidisciplinary (i.e., people or groups from different disciplines working together on research projects by drawing on their specific disciplinary knowledge) and interdisciplinary (i.e., synthesising methods and knowledge from different disciplines to answer research questions) approaches are needed to realise the full potential of these data [4]. Thus, over the last 15 years, epidemiologists have increasingly learnt the theories and language of colleagues from diverse fundamental and emerging disciplines, including mathematics, biology, chemistry, data and computer science and (bio)informatics [15–17]. We have worked in large collaborations with these disciplines, as well as with social and clinical scientists, with whom we have a long tradition of working. This multidisciplinary and interdisciplinary work with population data has been called ‘population health science’ [4].

### Causality, Mendelian randomisation and triangulation

One of the most notable changes in epidemiology in the last 15 years has been the increased use of Mendelian randomisation (MR) [18]. MR is the use of genetic data to explore causal effects of modifiable (non-genetic) risk factors. The first formal proposal of this method (as used over the last 15 years) was published in February 2003 [18], just 9 months before the first volume of *BMC Medicine* was published. Notably, in that original paper – and particularly in a subsequent paper – George Davey Smith acknowledges a long history of others who have suggested the use of genetic variants in this way, including Fisher, who made the link between randomised trials and the random segregation of genetic variants in 1951 [19]. MR and other new methods have stimulated debates about causality, the underlying assumptions of different analytical methods and the importance of acknowledging and exploring these [8]. This has resulted in epidemiologists increasingly using DAGs to demonstrate their causal analysis assumptions, particularly for new methods or causal frameworks, such as MR. Over the last 15 years, MR has been increasingly used to improve causal understanding of the effects of lifestyle risk factors and pathophysiological targets on human health and disease [20–24]. Alongside these applications, considerable efforts have been made to develop methods to explore the validity of the genetic instruments used in MR studies and the robustness of their results [25–34]. The availability of summary results from large numbers of genome-wide association studies (GWAS) that can be used for two-sample MR [29], together with automated tools (such as MR-Base [35]) for analysing these data and performing sensitivity analyses, have contributed to recent increases in the use of (two-sample) MR. This shift is reflected in the results of my review of *BMC Medicine* publications: just one MR study was published before 2018. This paper, published in 2004, did not use the term MR, but used *MTHFR* genetic variants to explore the role of homocysteine in migraine [36]. By contrast, six MR studies were published in *BMC Medicine* in 2018 [37–42], five of which used two-sample MR.

The ease with which two-sample MR can be undertaken means that some authors can complete analyses in minutes without giving sufficient thought to the importance or relevance of the research question being explored. They may also fail to consider or discuss key methodological issues (even when using automated systems developed specifically for two-sample MR). These include whether the two samples are from the same underlying population and whether the GWAS population used is relevant for the research question. In addition, replication of these two-sample MR findings and triangulating them with results from other methods with different underlying assumptions should be explored [29]. One notable example of the poor science that can result from the rush to an ‘easy publication’ is demonstrated by the comparison of results from two studies published in 2016. Both studies applied two-sample MR to the same publicly available data, but reported diametrically opposing conclusions (one reported that higher circulating C-reactive protein concentration increased risk of schizophrenia, while the other concluded that it decreased schizophrenia risk) [28]. Hartwig and colleagues demonstrated how one of the two had not harmonised summary data across the two samples (Table 3 in [28]); that paper has subsequently been retracted [43].

The use of triangulation is increasingly recognised as key to exploring causal effects [44]. In this approach, results are compared from several different epidemiological methods, each of which has different, unrelated, key sources of bias. The idea is that if each of these methods suggests that a risk factor is causally related to an outcome, despite their different sources of bias, confidence in the results increases and a true causal effect is reflected. If results differ, by being explicit in the first instance about their different sources of bias, it is possible to determine what further studies would be needed to obtain a robust causal answer [44]. Going forwards, the potential for further extending this approach in a truly interdisciplinary way – including integrating data from (bio)informatics and laboratory science – is an exciting possibility for the next 15 or more years.

### Data sharing and supporting team science

Changes in epidemiology over the last 15 years have coincided with debates about data-use and sharing [45]. In cohort studies, there is no equivalent of the randomised trial register that provides a means of exploring ‘data dredging’ and publication bias. In a 2007 commentary, I noted that with the increasing number of cohorts and data within them that are, rightly, shared across the global scientific community to investigate many different hypotheses, it was nearly impossible to judge contributions to publication bias from observational epidemiology [46]. I suggested then that this situation might be improved by changing the journal publication process so that authors submitted only the introduction and methods of their study. In this way, decisions to publish would not be dependent on the results (and whether or not they reached some arbitrary *P*-value threshold). This opinion had no influence on journal editors or researchers and, in fact, my thoughts have changed since then. I think accessing cohort data would benefit from the requirement to submit a brief ‘protocol’ of planned analyses that could serve as a ‘register’. These should be kept as simple as possible and made public. They should neither be used to judge (scientifically) whether data are shared, nor to reject access on the basis of overlap with other proposals. Two UK examples of this process are the UK Biobank and the Avon Longitudinal Study of Parents and Children (ALSPAC) [47, 48] (for transparency, I acknowledge that I have had a leading scientific role in ALSPAC for the last 15 years). Debates about the pros and cons of this approach versus access that does not require registration are likely to continue, but I hope over the coming years that more researchers, funders, academic institutions and journal editors will insist on clear policies for sharing of hypotheses, data and analysis code between researchers. In addition, they should push for ‘team science’, with recognition of all who contribute (including those who recruit participants and collect and process data).

## Conclusions

As a new member of the *BMC Medicine* Editorial Board, I am pleased to see that a consistently high proportion of applied epidemiology papers have been published over the last 15 years (Fig. 1a). As I read through the titles and abstracts of each paper, I also sensed that a high proportion of this research is from low and middle income countries, which I am also pleased about. In the next 15 years, it would be nice to see the advice to researchers from a recent *Nature* editorial reflected in published *BMC Medicine* research: ‘In short, be sceptical, pick a good question, and try to answer it in many ways. It takes many numbers to get close to the truth’ [49].

## Additional file

Additional file 1: Details of how the articles included in Fig. 1 were classified. (DOCX 15 kb)

## Data Availability

Not applicable.

## Abbreviations

GWAS: Genome-wide association study
MR: Mendelian randomisation
