# Supplementary material for: Fifteen years of epidemiology in BMC Medicine
Source: BMC Med. 2019 Sep 23;17:177. doi: 10.1186/s12916-019-1407-5 (PMC6755685; doi:10.1186/s12916-019-1407-5)
Supplement: Supplementary file 1 — Details of how the articles included in Fig. 1 were classified. (DOCX 15 kb) [file 12916_2019_1407_MOESM1_ESM.docx]

Additional file 1. Review of publications in *BMC Medicine* over the last 15 years: methods used to produce Figure 1.

I looked at the titles and abstracts of all *BMC Medicine* publications published between 2003 and 2018. I also read some papers more fully and/or searched for particular words (e.g., ‘Mendelian’) to aid classification.

I applied the following definitions to classify publications as research articles, epidemiological research, epidemiological methodological research, or an epidemiological research article in which the main analyses included one or more ‘omics measure:

- Research articles were defined as those labelled ‘research article’ by *BMC Medicine*
- Epidemiology research was broadly defined to include cross-sectional, case-control, cohort or randomised trials of defined populations (including general populations, clinical populations, children, the elderly, pregnant women, etc.) and systematic reviews of those studies [5]. It also included methodological research (see below), qualitative research, animal studies and research that analysed human tissue or cells only, along with several studies of associations between doctors’ success in university and specialist exams and some that were clinical audits (i.e., comparing observed performance against clinical guidelines).
- Epidemiological methods research was defined as publications describing the development or application of epidemiological study design methods, analysis methods, or data collection methods. Only articles that had been selected as ‘epidemiology research’ could be considered as epidemiological methods research.
- Any identified epidemiology research article was categorised as an ‘omics epidemiology paper if the main analyses included any of the following data types: genomic, epigenomic (including transcriptomic), metabolomic, proteomic or microbiomic data. Genomic and epigenomic data could be candidate or genome-wide.
- Mendelian randomisation (MR) studies were defined as any study that used genetic data to explore causal effects of a (non-genetic) modifiable risk factor, irrespective of whether authors used the term MR.

I went through each issue (year) online and kept a tally under each of the above defined article types. I then randomly selected 5 years (excluding 2003 given that it only included three articles): 2004, 2006, 2010, 2013, 2018) and repeated categorising and tallying – all numbers were identical or within three digits of the original. Where there were discrepancies, I took the average of the two rounded down to a whole integer.

I acknowledge that other authors may not have agreed with my classification of epidemiological papers.
